# Social anxiety in Finnish adolescents from 2013 to 2021: change from pre-COVID-19 to COVID-19 era, and mid-pandemic correlates

**DOI:** 10.1007/s00127-023-02466-4

**Published:** 2023-04-24

**Authors:** Klaus Ranta, Terhi Aalto-Setälä, Tiina Heikkinen, Olli Kiviruusu

**Affiliations:** 1 Department of Psychology, Tampere University, Tampere, Finland; 2 Department of Psychiatry, University of Helsinki, Helsinki, Finland; 3 Finnish Institute for Health and Welfare, Helsinki, Finland

**Keywords:** Social anxiety, Adolescents, COVID-19, Population epidemiology, Time trend

## Abstract

**Purpose:**

Social anxiety disorder (SAD) is prevalent in adolescents. Increase in levels of general anxiety since 2010’s has been observed in young people. Little is known of time trends in symptoms of social anxiety during 2010’s, of pre- to during-COVID-19 era changes, or of associations between social anxiety symptoms and pandemic severity, distance education, and COVID-19-related experiences in young people.

**Methods:**

We examined social anxiety symptoms, their temporal changes, and their associations with COVID-19 related factors in a sample of 450 000 13-to-20-year-old Finns in 2013–2021. Data from nationwide School Health Promotion study was used. Social anxiety symptoms were assessed with the Mini-SPIN using cut-off score ≥ 6 as indicator of high social anxiety. Multivariate logistic regression analyses were used, controlling for gender, age, family SES, and symptoms of general anxiety and depression.

**Results:**

High-level social anxiety symptoms increased markedly from 2013/2015 to 2021 among both sexes. A steeper increase was found among females. In 2021, 47% of females self-reported high social anxiety, a two-fold increase relative to 2013/2015. No association between regional COVID-19 incidence and change in social anxiety symptoms was found. No clear associations between time spent in distance education and social anxiety symptoms were found. Fears of getting infected or transmitting coronavirus, and reports of not getting needed support for schoolwork during distance education were all associated with high social anxiety.

**Conclusion:**

Prevalence of high social anxiety in young people aged 13–20 has increased considerably from 2013 to 2021, especially among girls. During COVID-19 pandemic, socially anxious young people report a need for educational support and suffer from infection-related fears.

**Supplementary Information:**

The online version contains supplementary material available at 10.1007/s00127-023-02466-4.

## Introduction

Social anxiety and its clinical form, social anxiety disorder (SAD) typically onset during adolescence. Epidemiological studies indicate that the highest period for incidence of SAD is between ages 10 and 19, and that higher prevalence is found among females [1–3]. Lifetime prevalence estimates for SAD in adolescent samples across countries range from 3 to 11% [4]. A Finnish study found a 12-month prevalence of 3.4% for SAD among 12-to-17-year-olds based on clinical interview [5]. Studies have mainly found social anxiety symptoms and SAD still increasing from adolescence into young adulthood [6, 7].

Symptomatic recovery from SAD without treatment seems more infrequent than in other anxiety disorders [8]. Social and educational impairments associated with high social anxiety increase in adolescence relative to childhood [9, 10]. Relative to their peers, youth with SAD have fewer close friends and dating partners [11, 12], and show slower academic progression and less successful transition to higher education [13, 14].

Few studies have examined secular changes in adolescents’ SAD symptoms or high social anxiety. Available studies suggest increase in adolescents’ and young adults’ social anxiety during the 2010’s compared with preceding decades, stronger for females under 18 years of age [6]. A Finnish study using a population sample from one large city, found an increase of high levels of social anxiety from 2002–2003 to 2018–2019 among both sexes, more notably in girls [15]. To compare, several studies show increase in adolescents’ symptoms of general anxiety across the globe, more in females: continuing from 1990’s to 2010’s, through 2010 –2020, and further intensifying during the COVID-19 pandemic [16–18].

The COVID-19 pandemic from 2020 on, has vastly affected the lives of adolescents across the globe. Disease containment measures have included school closures, transition to distance education and lockdown of sport practices. This has set adolescents vulnerable to mental health challenges [19]. For young people with social anxiety, social distancing measures such as applied during pandemic might first provide temporary symptomatic relief. However, after prolonged involuntary social isolation, return to social interactions may increase social anxiety [20]. Two recent, small-scale surveys in Autralia, UK, and USA found evidence of this, effect being marked especially for adolescents and young adults [21, 22].

Population research on changes in social anxiety symptoms of young people from pre- to during-COVID-19 era is scant. Available studies suggest increases [23–26]. A study among 2990 Canadian early adolescents showed no increase in symptoms of social anxiety from pre-pandemic to during-pandemic school cohorts in 2019 and 2020; however somewhat higher level of fear of judgment by others, a typical fear in SAD was observed [23]. In an American longitudinal study of 451 adolescents and young adults, assessments after the pandemic onset showed increased social anxiety levels among both sexes; instead home confinement concerns, such as having no social life were associated with decreased levels of social anxiety [24].

In Israel, small, consecutive cohorts of socially anxious, first year university students were followed up each year. Cohort 2019/2020, for whom COVID-19 social distancing measures were introduced at the end of Fall term, was compared with preceding year cohorts. While preceding cohorts showed decline in social anxiety symptoms over the academic year, for 2019/2020 cohort symptoms remained high [25]. In China, using population-validated measures, social anxiety symptoms during the COVID-19 pandemic were found to be higher than national norms among a predominantly female university student sample (*N* = 3137) [26].

To sum, practically no studies, either in Finland or internationally, have examined time trends, pre- to during-COVID-19 change, its moderators, and pandemic-related correlates related to social anxiety symptoms in adolescents and young adults using large, representative population samples and validated symptom measures. This constitutes a gap in the current research.

The first aim of the present study was to examine the prevalence of social anxiety symptoms across gender and age groups from years 2013 to 2021 among Finnish young people aged 13–20 years. Second, we examined pre- to during-COVID-19 change in SAD symptoms and the moderating effects of gender, age, and geographic region/regional COVID-19 incidence on this change. Third, we studied cross-sectional associations between distance education, support given for schoolwork, infection and transmitting fears, and changes in social contacts, and social anxiety symptoms during the pandemic in Spring 2021. We used the nation-wide School Health Promotion Study (SHP) data, with over 450 000 participants across the study years [27].

Our hypotheses were: 1. Social anxiety symptoms in 2013–2021 will likely be more frequent than symptom-based estimates from years before 2013, possibly more in girls, and in over 18 -year-olds; 2. Increase in social anxiety symptoms will likely be observed from pre-to during COVID-19 era; 3. Distance education may either decrease or elevate social anxiety symptoms, and among the socially anxious, perceived support for schoolwork will be low, infection and transmitting fears will be elevated, and social contacts will be reduced.

## Method

### Subjects and procedure

The Finnish School Health Promotion (SHP) study is a nationwide survey conducted biennially since 1996. It is based on total sampling. All Finnish municipalities arranging primary or secondary level education are invited to participate. The SHP gathers data on well-being, health, mental health, schoolwork, and life circumstances of 8th and 9th students in the comprehensive schools, and 1st and 2nd year students in high schools and vocational schools. Subjects complete the SHP questionnaire independently and anonymously during a school lesson, taking the whole lesson. In 2021, the survey online form included 108 items with multiple response alternatives. Responding to the survey is voluntary. The SHP research plan has been evaluated by the institutional review board of the Finnish Institution for Health and Welfare [27].

The present study uses data from years 2013, 2015, and 2021, when measure of social anxiety symptoms, the Mini-SPIN [28] was included in SHP. Coverage rates in 2021 were 75% (*N* = 91 560) in comprehensive school, 71% (*N* = 47 383) in high school, and 32% (*N* = 21 853) in vocational school [27, 29].

### Measures

All data were based on self-reports of the students, except information about region of Finland and regional COVID-19 incidence rate.

### Demographic and background variables

Questions relating to age, gender, demographic and socioeconomic background factors, and immigrant status are described in SHP publications [27, 29].

Respondents reported their official gender. Mother’s and father’s highest education was queried with four answering categories: 1. basic compulsory; 2. upper secondary or vocational; 3. upper secondary/vocational + additional vocational studies; 4. university or university of applied sciences. For the analyses, a two-category variable “Higher parental education” (yes/no) was formed indicating that at least one parent had a university degree. Regarding living arrangements, respondents were asked whether they were living in a household with both parents (yes/no). Immigrant status/family origin was based on respondent’s own and their parents’ country of birth. A four-category variable was formed: 1. Finnish origin; 2. one parent of foreign origin; 3. both parents of foreign origin, participating adolescent born in Finland; 4. both parents and participating adolescent of foreign origin.

Geographic regions were classified as divided into seven Finnish Regional State Administrative Agencies [30]. These agencies are in charge of deciding most of the COVID-19-related regional disease containment measures. The severity of regional pandemic, based on COVID-19 incidence rates differed between the regions. Incidence rate of COVID-19 infections was calculated for each region for the education year 2020–2021 (1st Aug 2020 to 31st May 2021) per 100 000 inhabitants (population at 31st Dec 2020). COVID-19 incidence data was retrieved from the Finnish National Infectious Diseases Register [31].

### Social anxiety symptoms

The Mini-SPIN [28] is a three-item instrument for detecting symptoms of SAD. The items are: 1. “Fear of embarrassment causes me to avoid doing things or speaking to people”; 2. “I avoid activities in which I am the center of attention”; and 3. “Being embarrassed or looking stupid are among my worst fears”. Respondents rate how much they have been bothered by these problems during the preceding week on a five-point scale. Mini-SPIN has shown validity to identify social anxiety symptoms warranting clinical attention among adolescents and young adults in European studies [32, 33]. In a Finnish study, Mini-SPIN showed a sensitivity of 86%, specificity of 84%, and positive and negative predictive values of 26% and 99% for detecting SAD in adolescents using a cut-point of six points or more [32]. The cut-point of six was used in the present study to indicate high level of social anxiety [32, 33].

### Symptoms of generalized anxiety and depression

The GAD-7 [34], a seven-item instrument was used as measure of generalized anxiety disorder (GAD) symptoms. Items cover the DSM-IV symptoms of GAD. A cut-off score of 10 points or more was used to reflect at least moderate levels of generalized anxiety, as used among young adults and adolescents [34, 35]. The Finnish translation of GAD-7 has shown acceptable psychometric properties among adolescents [36]. Symptoms of depression were assessed with PHQ-2, a two-item depression measure [37]. It covers anhedonia and low mood, two key symptoms of DSM-V major depressive disorder. The cutpoint of 3 was used, as suggested for adolescents and adults [37, 38].

### Questions on COVID-19 pandemic and everyday life

In 2021 SHP survey included questions related to COVID-19 pandemic, covering distance education arrangements, worries related to coronavirus, and contacts with friends [27, 29].

The participants were asked about whether they had received distance education. If the answer was affirmative, they were asked time in months spent in distance education during past Fall and Spring terms. To reflect the whole school year, a four-category variable was formed: 1. less than one month per term; 2. 1–2 months per term at most; 3. over two months in either term; 4. over two months in both terms.

To measure perceived support for schoolwork during distance education participants were asked: “During this school year, have you received support and help for learning and schoolwork at distance teaching?” The response alternatives were: 1. yes, a lot; 2. yes, some; 3. no, but I would have needed it; 4. I have not needed any help; 5. I have not had distance teaching. A three-category variable was formed: 1. not needed / received a lot; 2. received some; 3. not received, would have needed. Affirmative responses to alternative 5 (I have not had distance teaching) were coded as missing/excluded, as the interest was in support received during distance education.

To assess worries related to COVID-19 infection participants were asked: “People may have concerns about coronavirus. Have you been worried about any of the following things during this school year?” Thereafter, a list of items was presented, including: “Getting infected with the coronavirus” and “That you may infect others”. Response alternatives were: 1. not at all; 2. to some extent; 3. a lot.

To assess contacts with friends, participants were asked whether the coronavirus epidemic, or the restrictions caused by it had affected keeping in contact with friends during the past school year. Response alternatives were: 1. no influence; 2. yes, decreased; 3. yes, increased; 4. not applicable to me. Affirmative responses to alternative 4. were coded as missing, and thus excluded from the analyses.

### Statistical analyses

For the most, males and females were analysed separately. No missing values were allowed for gender and Mini-SPIN, resulting in exclusion of 10 104 responses (2.2%), 453 956 responses remaining in the analysed sample. Regarding other study variables, percentages of missing data are presented in Appendix Tables 1, 2, 3.

**Table 1:** Percentages and 95% Confidence Intervals (95% CI) of those with high social anxiety (Mini-SPIN ≥ 6) among 13 to 20-year-old Finns in years 2013, 2015 and 2021 by gender and age group

	2013		2015		2021		Total
	% (95% CI)		% (95% CI)		% (95% CI)		% (95% CI)
Females, total	24.2 (23.9–24.5)		26.4 (26.1–26.7)		46.9 (46.6–47.2)		32.7 (32.5–32.9)
Females by age group							
13–15 years	25.9 (25.5–26.3)		26.7 (26.1–27.3)		48.4 (47.9–48.9)		34.8 (34.5–35.1)
16–17 years	23.3 (22.9–23.7)		26.5 (26.0–27.0)		46.3 (45.8–46.8)		31.9 (31.6–32.2)
18–20 years	21.4 (20.6–22.2)		24.9 (24.0–25.8)		43.1 (42.0–44.2)		28.7 (28.2–29.2)
Males, total	14.2 (14.0–14.4)		16.2 (15.9–16.5)		21.3 (21.0–21.6)		17.1 (16.9–17.3)
Males by age group							
13–15 years	14.0 (13.7–14.3)		15.4 (14.9–15.9)		21.0 (20.6–21.4)		16.9 (16.7–17.1)
16–17 years	14.4 (14.1–14.7)		16.6 (16.2–17.0)		21.5 (21.1–21.9)		17.3 (17.1–17.5)
18–20 years	14.2 (13.5–14.9)		17.2 (16.3–18.1)		22.5 (21.4–23.6)		17.4 (16.9–17.9)

**Table 2:** Effect of COVID-19 pandemic (year 2021) on social anxiety (Mini-SPIN ≥ 6) among 13 to 20-year-old Finns compared to pre-pandemic levels (years 2013 and 2015) in females and males

		Females			Males	
		OR (95% CI)	*p*		OR (95% CI)	*p*
Models comparing to pre-pandemic levels^2^						
Total						
COVID-19 (year 2021)^3^		2.69^A^ (2.64–2.74)	< 0.0001		1.56^A^ (1.52–1.60)	< 0.0001
Age groups						
13–15 years						
COVID-19 (year 2021)^3^		2.71 (2.63–2.79)	< 0.0001		1.57 (1.51–1.62)	< 0.0001
16–17 years						
COVID-19 (year 2021)^3^		2.69^b^ (2.62–2.77)	< 0.0001		1.55^b^ (1.49–1.60)	< 0.0001
18–20 years						
COVID-19 (year 2021)^3^		2.61^b^ (2.46–2.77)	< 0.0001		1.61^b^ (1.49–1.74)	< 0.0001
Model with linear time trend estimate included^4^						
Total						
Time since 2013, years		1.06^a^ (1.05–1.08)	< 0.0001		1.07^a^ (1.06–1.09)	< 0.0001
COVID-19 (year 2021)^3^		1.73^A^ (1.58–1.89)	< 0.0001		0.94^A^ (0.84–1.05)	0.2949

Odds ratios (OR) and 95% Confidence Intervals (95% CI) from adjusted^1^ logistic regression analyses by age group

^1^All models adjusted for age group (total models only), parental education, living with both parents, immigrant status and region (for unadjusted models and models adjusted for generalized anxiety, see Appendix Table 2)

^2^Year 2021 compared to years 2013 and 2015 combined

^3^Coded “1” for year 2021, “0” for years 2013 and 2015

^4^In addition to the COVID-19 parameter (year 2021) also a parameter for linear time starting from year 2013 included

Difference in effects between genders: ^A^ significant (*p* < 0.0001); ^a^ non-significant (*p* > 0.05)

Difference in effects between age groups (within genders comparing to 13–15-year-olds): ^b^ non-significant (*p* > 0.05)

**Table 3:** Regional COVID-19 incidence rates, prevalence of social anxiety (Mini-SPIN ≥ 6), and Odds ratios (OR) for COVID-19 outbreak on social anxiety among females and males

Region	COVID-19 incidence rate^1^			Prevalence of social anxiety in 2021			Effect of COVID-19 on social anxiety^3^	
	Per 100,000	Rank^2^		%	Rank^2^		OR (95% CI)	Rank^2^
Females								
Eastern Finland	557	1		47.4	5		2.81 (2.65–2.98)	6
Lapland	580	2		48.2	7		2.40 (2.17–2.66)**	1
Northern Finland	679	3		47.5	6		2.46 (2.33–2.60)**	2
Western and Inland Finland	881	4		46.0	2		2.62 (2.52–2.72)**	5
Åland	1207	5		39.3	1		2.46 (1.90–3.19)	3
Southwestern Finland	1595	6		46.9	3		2.54 (2.41–2.67)**	4
Southern Finland	2343	7		47.2	4		2.86 (2.78–2.95)	7
Males								
Eastern Finland	557	1		20.3	3		1.52 (1.41–1.64)^#^	4
Lapland	580	2		23.5	7		1.59 (1.39–1.80)	6
Northern Finland	679	3		21.4	4		1.43 (1.33–1.53)**	2
Western and Inland Finland	881	4		20.1	2		1.50 (1.42–1.57)**	3
Åland	1207	5		17.5	1		1.26 (0.91–1.75)	1
Southwestern Finland	1595	6		22.4	6		1.53 (1.43–1.63)^#^	5
Southern Finland	2343	7		21.9	5		1.67 (1.61–1.74)	7

^1^All COVID-19 incidences between 1.8.2020 and 31.5.2021 divided by the total population in that area * 100,000

^2^Rank based on values in the preceding column in ascending order

^3^Logistic regression models adjusted for age group, parental education, living with both parents and immigrant status

^#^*p* < 0.05, **p < 0.001—significance of the difference in effects comparing to Southern Finland

Associations between gender, age group and social anxiety symptoms were analyzed using logistic regression (LR) modelling. Odds ratios (OR) with 95% confidence intervals (CI) were calculated. Gender differences in the effect of age group to SAD symptoms were tested using gender x age group interaction terms.

Percentages (95% CI) of participants with high level of social anxiety were calculated for each study year. Change in the prevalence of high social anxiety from pre- to during-COVID-19 time was analysed also using LR models. The COVID-19 outbreak was defined in LR by assigning value “1” for year 2021 and value “0” to otherwise, resulting in a dichotomous variable. Thus year 2021 was compared against years 2013 and 2015 combined. Additionally, a model including a parameter for continuous time (in years) beginning from year 2013 was fitted, where the effect of COVID-19 (year 2021) was interpreted as the deviation between values observed in 2021 and those projected for 2021 based on the pre-pandemic trend from years 2013–2015.

LR models were run separately for females and males, and within genders also by age group and by region. Differences according to these factors in pre- to during-COVID-19 social anxiety symptom changes i.e., whether they moderated this effect, were analyzed using interaction terms. The LR models examining temporal change were first run without control variables (i.e., unadjusted), then adjusted for age group, parental education, living with both parents, immigrant status/family origin, and region. Results from the adjusted LR models are reported in the main article (Table 2). Results from unadjusted LR models and models additionally adjusted for general anxiety on pre- to during-COVID-19 SAD symptom change are presented in Appendix Table 2.

A descriptive analysis across geographic regions was performed examining pre- to during-COVID-19 social anxiety symptom change displayed against regional COVID-19 infection incidence rates.

Cross-sectional associations between variables assessed during COVID-19 pandemic (covering experiences of distance education, fears related to infection, contacts with friends) and social anxiety symptoms in 2021 were also analyzed using LR. In these models, in addition to above-mentioned adjustments, depressive symptoms and GAD symptoms were also controlled for. From year 2021 analyses, 845 (0.5%) cases were excluded due to implausible responding [39]. All analyses were done using SPSS 26.0 software.

## Results

### Descriptive statistics by study year

Descriptive statistics of the sample and study variables by study year are given in Appendix Table 1. Some changes during the period were observed. In 2015 the number of participants was lower compared to other years. In 2015, the relative proportion of participants in 8th and 9th grades was lower compared to other years. Compared to years 2013–2015, the number of parents with higher education was higher in 2021.

### Gender and age differences in social anxiety symptoms

Across the three study years the percentage of participants with high level of social anxiety symptoms was 32.7% in females and 17.1% in males (Table 1). The OR for female gender was 2.36 (95% CI 2.33–2.40) in the adjusted LR model.

Considering the effect of age, prevalence of high social anxiety decreased with increasing age across the three study years among females. The prevalence was 35% among 13–15-year-olds and 29% among 18–20-year-olds (Table 1). Among males, prevalence showed less variation by age group, although indicating slightly increasing prevalence of high social anxiety with increasing age. The divergent age gradients between genders were confirmed by observing statistically significant gender × 16–17-year-olds (*p* < 0.0001) and gender × 18–20-year-olds (*p* < 0.0001) interaction effects on social anxiety symptoms in LR models with 13–15-year-olds as the reference group.

### Secular change in social anxiety symptoms and the effect of COVID-19 pandemic

**Fig. 1:**
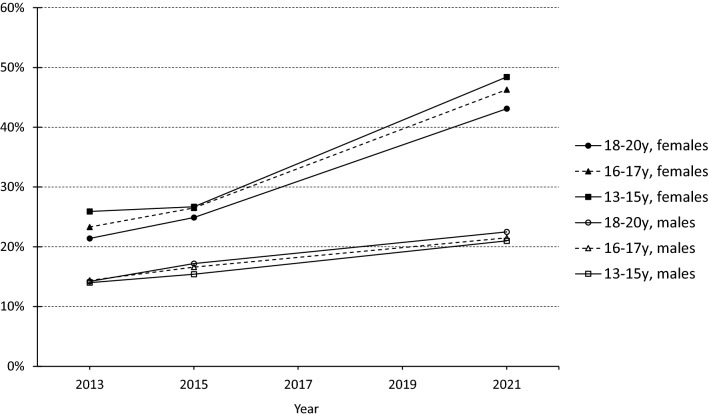
Percentages of participants with high-level social anxiety symptoms (Mini-SPIN ≥ 6) among 13 to 20-year-old Finns from year 2013 to 2021 by gender and age group

Among females, proportion of participants with high social anxiety was around 25% in 2013 and 2015, but in year 2021 it had gone up to a level as high as 47% (Table 1, Appendix Fig. 1). Among males the corresponding figures were around 15% for years 2013 and 2015 and 21% in 2021. In adjusted LR models the OR of COVID-19 (i.e., year 2021) on high level of social anxiety symptoms was 2.7 in females and 1.6 in males compared to pre-pandemic prevalence (years 2013 and 2015 combined) (Table 2). The larger effect in females compared to males was statistically significant (*p* < 0.0001) as analyzed using COVID-19 × gender interaction term. There were no significant differences between the age groups in the effect of COVID-19 on the prevalence of high level social anxiety symptoms either among females or males (Table 2, Fig. 1).

The LR models adjusted for background factors are presented in Table 2. Comparisons to unadjusted LR models, and LR models additionally adjusted with GAD symptoms showed only modest or negligible differences in the estimates, with a small attenuation in the effect of COVID-19 on social anxiety symptoms in females after adjusting for GAD symptoms (see Appendix Table 2).

In addition, the effect of COVID-19 pandemic on high level social anxiety symptoms was modelled against projected trend of prevalence based on 2013 and 2015 social anxiety symptom prevalence (Table 2). The effect of time on the prevalence of social anxiety symptoms was positive with no significant gender differences. With the projected trend modelled, the effect of COVID-19 was significant in females (OR = 1.7), but not in males (OR = 0.9), again indicating a statistically significant gender difference in the effect of COVID-19 on social anxiety symptoms (Table 2, Appendix Fig. 1).

The regional prevalence rates of high social anxiety in 2021, and the effect of COVID-19 (i.e., year 2021 compared to years 2013 and 2015 combined) on social anxiety symptoms are displayed against regional rates of COVID-19 incidence during the school year preceding the survey in 2021 in Table 3. Although there were significant regional differences in the effect of COVID-19 on social anxiety symptoms, the figures sorted in ascending order of regional COVID-19 incidence indicate no meaningful associations between either regional COVID-19 incidence and prevalence of high social anxiety in 2021, or regional COVID-19 incidence and effect of COVID-19 on social anxiety symptoms.

### Experiences during COVID-19 pandemic and social anxiety symptoms

The cross-sectional associations between experiences during COVID-19 pandemic and high social anxiety symptoms in 2021 are presented in Table 4. Distributions of answers and gender differences for these measures are presented in Appendix Table 3. Time spent in distance education was associated with an increased risk of high social anxiety, especially if it lasted over 2 months in either Fall/Spring term, but the effects were small and further attenuated when adjusting for general anxiety and depressive symptoms. Participants who did not receive support they needed for learning while in remote school had an increased risk of high social anxiety, effect being more pronounced among males than females. The effects attenuated when symptoms of general anxiety and depression were controlled for, but remained statistically significant.

**Table 4:** Effects of COVID-19-related measures of SHP 2021 on symptoms of social anxiety (Mini-SPIN ≥ 6) among 13 to 20-year-old Finns in 2021 in females and males

COVID19-related measures in SHP 2021: effect of COVID-19 on everyday life	Adjusted model 1^1^				Adjusted model 2			
	Females		Males		Females		Males	
	OR (95% CI)	*p*	OR (95% CI)	*p*	OR (95% CI)	*P*	OR (95% CI)	*p*
Time spent in remote school								
Less than 1 month per semester (ref)	1.00		1.00				1.00	
1–2 months per semester at most	1.05 (1.01–1.09)	0.0123	1.03 (0.98–1.08)	0.2311	1.04 (0.99–1.08)	0.1004	1.02 (0.97–1.07)	0.4552
Over 2 months in either semester	1.09** (1.04–1.14)	0.0009	1.11**(1.05–1.19)	0.0010	1.04**(0.98–1.09)	0.1765	1.08**(1.01–1.15)	0.0274
Over 2 months in both semesters	1.07^#^ (1.00–1.15)	0.0421	1.03^#^ (0.95–1.12)	0.4671	0.94*(0.88–1.01)	0.0906	0.95*(0.87–1.03)	0.1881
Support received in remote school for studies/learning								
No need/received plenty (ref)	1.00		1.00		1.00		1.00	
Received some	1.04*** (1.00–1.07)	0.0469	1.16***(1.10–1.21)	< 0.0001	0.96***(0.93–1.00)	0.0396	1.11***(1.06–1.17)	< 0.0001
Not received, would have needed	1.63*** (1.56–1.70)	< 0.0001	1.93***(1.81–2.05)	< 0.0001	1.12***(1.07–1.17)	< 0.0001	1.35***(1.26–1.45)	< 0.0001
Worry, get infected, self								
Not at all (ref)	1.00		1.00		1.00		1.00	
Some	1.12*** (1.08–1.15)	< 0.0001	1.38***(1.32–1.43)	< 0.0001	1.13***(1.09–1.16)	< 0.0001	1.35***(1.29–1.41)	< 0.0001
A lot	1.57*** (1.49–1.66)	< 0.0001	2.06***(1.86–2.28)	< 0.0001	1.34***(1.26–1.42)	< 0.0001	1.63***(1.46–1.82)	< 0.0001
Worry, transmit to others								
Not at all (ref)	1.00		1.00		1.00		1.00	
Some	1.12*** (1.08–1.15)	< 0.0001	1.36***(1.31–1.42)	< 0.0001	1.09***(1.06–1.13)	< 0.0001	1.32***(1.27–1.38)	< 0.0001
A lot	1.59*** (1.52–1.66)	< 0.0001	2.05***(1.91–2.20)	< 0.0001	1.31***(1.25–1.38)	< 0.0001	1.66***(1.53–1.79)	< 0.0001
Effect on contacts with friends								
No effect (ref)	1.00		1.00		1.00		1.00	
Decreased	1.39*** (1.34–1.43)	< 0.0001	1.51***(1.44–1.58)	< 0.0001	1.23***(1.19–1.27)	< 0.0001	1.36***(1.30–1.42)	< 0.0001
Increased	1.22*** (1.17–1.28)	< 0.0001	1.50***(1.41–1.59)	< 0.0001	1.08***(1.03–1.13)	0.0021	1.33***(1.24–1.42)	< 0.0001

Odds ratios (OR) and 95% Confidence Intervals (95% CI) from adjusted logistic regression analyses

^1^Model 1: adjusted for age group, parental education, living with both parents, immigrant status and region; Model 2: in addition to model 1 adjustments, adjusted for generalized anxiety (GAD-7 ≥ 10) and being depressed (PHQ-2 ≥ 3)

Significance of the difference in effects between genders: *p < 0.05, *p < 0.01, **p < 0.001, ***p < 0.0001

Participants who worried a lot of getting coronavirus infection or transmitting it to others, had an increased risk of high social anxiety, showing also in adjusted LR models, in which symptoms of GAD and depressed were controlled for. Effects were again stronger for males as indicated by statistically significant gender × worry variable interaction terms on social anxiety symptoms. Finally, both perceived pandemic-related decrease and increase in contacts with friends were associated with higher odds of having high social anxiety. Among males the effects were of equal size, while among females the effect of decrease in contacts was stronger, especially in the model adjusted for general anxiety and depressive symptoms (Table 4).

## Discussion

Our results show a high prevalence of social anxiety symptoms exceeding the cut-off for high social anxiety among Finnish young people in 2013–2021, clearly higher among females than among males. In females, social anxiety symptoms decreased with age, in males they slightly increased. Social anxiety symptoms increased from pre- to during-COVID-19 era among both sexes, in females more than in males. There was no consistent association between regional COVID-19 incidence and pre- to during-COVID-19 change in high social anxiety symptoms. In 2021, a year after pandemic onset, number of months spent in distance education during past school year was only marginally associated with social anxiety, and time/anxiety association was not linear. Unmet need for support in schoolwork was associated with high social anxiety among both sexes, more strongly among males. Fears of getting infected and of transmitting others were associated with high social anxiety in both sexes, again more strongly in males.

### Social anxiety symptoms 2013–2021

The observed prevalence of high-level social anxiety symptoms, among males 14% to 21%, among females 24–47% during the study time span, is broadly in line with prevalence of social fears and symptoms of social anxiety found in surveys in other countries in adolescent and young adult populations [4, 6, 40, 41]. In comparison to years up to 2013, symptom rate shows an increase, accordant with our first hypothesis. A Finnish, smaller population study [15], found similar increase, and a similar gender pattern in the change of social anxiety symptoms, i.e., more pronounced among girls, but also significant in boys, from 2002–2003 to 2018–2019. Using Mini-SPIN and the same cutpoint, an Australian online survey reported a prevalence of 32% for high social anxiety symptoms in adolescents in 2019, prior to pandemic [40]. Obviously, prevalence rates based on self-reported social anxiety symptoms are considerably higher than those found for clinical SAD, ascertained with a clinical interview [5, 7, 42–44] due to well-known effects of instrument selection, definition of caseness, and used impairment criteria on prevalence [44, 45]. Assessing self-reported social anxiety symptoms in the population, even with validated measures, and empirically defined cut points, does not identify a group of individuals with SAD, rather a group of individuals with self-perceived symptoms, among whom the risk of clinical SAD is heightened.

That females reported higher social anxiety than males, is consistent with findings from surveys [4, 40, 41, 46], and findings from interview-based SAD studies [7, 43, 44] across countries. However, some surveys report equal levels of social anxiety symptoms [6, 47] and some interview studies report equal rates of SAD [5, 42] between the sexes. Likely both cross-cultural variation and methodological variation contribute to contrasting findings [4, 45], as even within same country choice between instrument assessing trait social anxiety vs. DSM-based SAD symptoms may reveal different male-to-female ratios [41, 47]. Examining age, we found symptom decrease with age in females, but fairly even, even slightly increasing prevalence rate in males from young adolescence to early adulthood. This contrasts with our first hypothesis, based on findings from studies reporting an increase in social anxiety symptoms for both genders into young adulthood [40], and those reporting an increase of SAD for both genders [7, 48]; but also with findings from studies reporting prevalence of SAD decreasing with age in males but increasing with age in females [44]. Clearly, more research in population samples covering both adolescents and young adults is needed to examine these gender and age interactions more closely.

### Change in social anxiety symptoms from pre- to during-COVID-19 time

The increase in high level social anxiety symptoms from pre- to during-COVID-19 time and for both sexes found in this study, is consistent with findings from studies conducted in USA, Israel, and China [24–26], and according to our second hypothesis. Important to note, pre-COVID-19 comparison assessments were from 2013/2015. It is possible that social anxiety symptom levels could have been higher in 2017–2019, resulting in a smaller or no change. That increase was clearly greater in females suggests that gender-specific factors might be involved. For adolescent girls, close relationships and support from these may be a particularly important shield against social anxiety [49]. As social isolation caused by COVID-19 measures increases, maintenance of relationships might become more difficult, and social interactions may be more uncertain. For socially anxious girls, this may create more pressure and discrepancy with social norms, as females self-construals are more interdependent than boys [50, 51].

However, it is important to consider factors having impact over the longer, 8-year time period. Such factors might include increasing use of social media, which in several cross-sectional and some longitudinal studies has associated to heightened social anxiety in young people [52–54]. Excessive social media use seems to associate to internalizing symptoms more in girls [55, 56]. It appears that COVID-19 restrictions have contributed to an even increasing use of social media in young people [57].

The results revealed no consistent associations between regional COVID-19 incidence and pre- to during-pandemic increase in high social anxiety. Even though symptom increase was highest in Southern Finland, where incidence of COVID-19 also was highest, examination of other six areas showed no systematic associations. One Chinese study [58] reported, that strong regional restrictive measures buffered the effect of regional COVID-19 incidence on anxiety. However, this study used a different conceptualization of social anxiety.

### Experiences during the pandemic

#### Distance education

Time spent in distance education might be predicted to associate with heightened social anxiety, as schools are important socialization environments [20]. In this study, young people retrospectively reported time in distance education during the previous school year. Results showed few, modest associations to high social anxiety and no linear correlation between time in distance education and social anxiety, being consistent with our prediction that these associations may show in either direction. Although socially anxious adolescents may prefer online communication environments [59], associations between involuntary social isolation and social anxiety may be unpredictable. The effects of social anxiety may be greatest when students return to school [21]. It is conceivable, that alternating periods of involuntary distance education and normal school during the COVID-19 pandemic are not causing clear-cut effects to social anxiety.

#### Support for schoolwork

Our finding of association between perceived unavailability of support to schoolwork when needed, and high social anxiety was expected and might relate to findings on socially anxious adolescents’ multiple problems in educational functioning and in communicating their needs for support to adults, such as teachers [60]. Expressing need for support might be even more difficult and complicated during COVID-19 pandemic, and yet continued support may be especially important for these adolescents. Research suggests that social support not only reduces risk of social anxiety in adolescents, but it might also mitigate effects of negative life events on social anxiety symptoms among them [61]. Interestingly, recent findings suggest that need for social support among socially anxious may even be greater during involuntary social distancing [62]. Continuing educational support likely represents an important dimension of support for these young people.

#### Infection-related worries

Fear of getting infected was, as expected, associated with high-level social anxiety symptoms among both genders in this study. This finding concurs with findings of higher levels of COVID-19 fears among young adults with SAD compared with controls [63]. Fear of transmitting the virus has been suggested to cause major stress for adolescents [64]. Indeed, this fear was associated with high social anxiety in this large sample of Finnish young people among both sexes. Although greater conformity to social norms might be more typical for the female gender role and result in fears of closed ones getting infection [65], the social conformity tendency and fear of negative evaluation resulting from perceived breaching of norms (i.e., being recognized as the one who transmitted) is likely involved in social anxiety among both sexes [66]. Our results suggest that association between high social anxiety and infection and transmission fears are distinct to social anxiety, independently from gender-associated concerns, or illness fears related to general anxiety [67], controlled for in this study.

#### Contacts with friends

The finding that high social anxiety was associated with perceived pandemic-related change in contacts with friends, among both those who perceived decrease and those who perceived increase, was unexpected, but may reflect the complexity of associations between social anxiety and social functioning during the pandemic [62, 68]. Schools may represent contexts in which social interactions arise naturally, also for the socially anxious, while during social distancing, alternative contacting channels such as social media may pose new challenges. Unfortunately, due to item wording, we couldn’t distinguish between face-to-face and online contacts in this study. Thus, excessive use of social media for keeping contact with friends could have affected the results [52–54].

Some limitations of this study should be noted. First, we could not use a continuous biennial time series of social anxiety measurements from 2013 to 2021. This affects the reliability of time trend estimation. Due to absent data from 2017 and 2019, only rough estimate is possible. As there is some evidence of social anxiety symptom increase 2018–2019 relative to 2002–03 [15], and nearly as high symptom prevalence in Australia in 2019 [40], there remains a possibility that at least partially the symptom increase might have occurred during the last years of 2010’s, i.e., during pre-COVID-19 era. Response rate was low in vocational schools. This limits the representativeness and generalizability of the results in the age group of 16–20 years, and may cause bias, given social anxiety associates with compromised school attendance [12, 14]. Use of self-report reveals only partial view on social anxiety and does not replace clinical assessment. We could not use techniques such as counterbalancing the presentation order of measures in order diminish the effect order on the results. For further reference, see link to survey forms in English [69].

This study has also notable strengths. The SHP database is nationally representative, large population sample of Finnish adolescents. Main psychopathology measures are validated in the Finnish adolescent age group, and the effect of main comorbidity for social anxiety (i.e., depression and GAD symptoms) [42] was controlled for.

Some findings merit further study. Secular changes in social anxiety symptoms in combined adolescent and young adult samples is an important target for future studies. Gender-related factors associating with changes in social functioning among socially anxious youth during involuntary social isolation, both in real-life and online environments requires more study. Research into disorder-specific cognitive factors associated with social anxiety and pandemic-related social impairment seems important. Practical implications for professionals working with adolescents include acknowledging the impact of pandemic to the possible increase of social anxiety symptoms, particularly among girls; that socially anxious students may require extra support for schoolwork during distance education; and that discussing both fears of getting infected and transmitting virus are indicated in their work with socially anxious students.

To conclude, high level social anxiety symptoms showed a clear increase from 2013 to 2021 among Finnish young people. The increase was clearly more marked in females. During the pandemic unmet need for support in schoolwork, and fears of getting COVID-19 infection, or transmitting the virus were all associated with high social anxiety.

### Electronic supplementary material

Below is the link to the electronic supplementary material.

Supplementary file1 (DOCX 36 kb)

## Figures and Tables

**Fig. 2:**
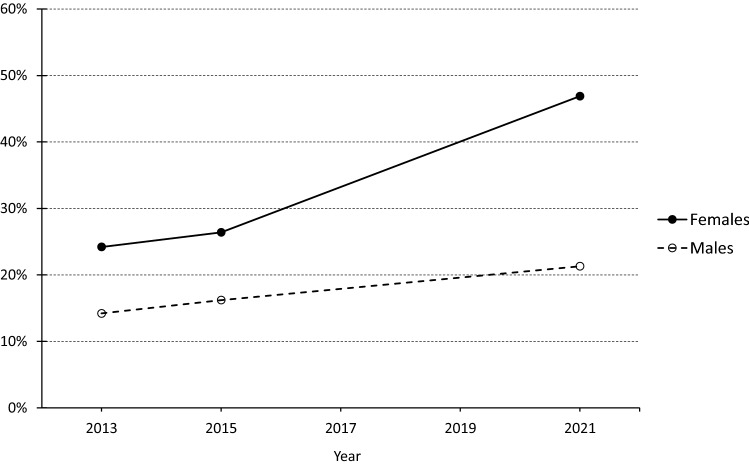
Percentages of those with clinical level social anxiety disorder symptoms (Mini-SPIN ≥ 6) among 13 to 20-year-old Finns in years 2013, 2015 and 2021 by gender

## Appendix

See Tables 5, 6, 7 and Fig. 2.

**Table 5:** Study participants and descriptive statistics of the study variables by study year, %

	Missing data^1^		2013 *N* = 178,706	2015 *N* = 118,772	2021 *N* = 156,478
Socio-demographic variables					
Gender	0.1/0.0				
Female			51.0	51.7	52.8
Male			49.0	48.3	47.2
Age group	0.2/0.0				
13–15 years			43.3	33.7	46.4
16–17 years			45.9	52.8	44.9
18–20 years			10.8	13.5	8.6
Higher parental education^2^	4.8/4.3		46.3	45.7	56.6
Living with both parents	2.9/2.5		65.3	63.7	67.1
Immigrant status/family origin	2.5/2.1				
Finnish origin			90.2	89.0	87.0
One parent of foreign origin			5.7	6.2	7.4
Foreign origin, born in Finland			1.5	1.7	2.2
Foreign origin, not born in Finland			2.7	3.1	3.4
Region of Finland^3^	0.0/0.0				
Southern Finland			39.2	39.2	38.8
Southwestern Finland			12.7	14.0	12.4
Eastern Finland			10.7	10.4	9.7
Western and Inland Finland			23.1	22.7	24.0
Northern Finland			10.1	10.3	11.3
Lapland			3.6	2.9	3.2
Åland			0.6	0.6	0.6
Psychiatric symptoms: control variables					
Generalized anxiety (GAD-7 ≥ 10)	1.0/0.3		10.9	11.6	19.6
Depressed (PHQ-2 ≥ 3)	2.2/1.4^4^				21.1
Psychiatric symptoms: outcome measure					
Social anxiety (Mini-SPIN ≥ 6)	1.9/0.0		19.3	21.5	34.8

^1^Of all participants (before slash) / of those fulfilling the inclusion criteria for the study (after slash)

^2^At least one parent with university degree

^3^Based on Regional State Administrative Agencies

^4^Considering year 2021 when PHQ-2 was available

Note: Information on age was missing from 6620 (1.4%) survey forms. These responses were coded with mode age of the grade the participant was in. Age reporting was unreliable (e.g., lower than 15 years in upper secondary education) in 850 (0.2%) forms. Responses were coded as missing information and thereby excluded from the analyses. Only 403 respondents aged 13 years in the age group of 13–15-year-olds. For years 2013, 2015, and 2021 in total there were 439 (0.1%) survey forms with missing information on official gender. They were excluded from the analyses

**Table 6:** Effect of COVID-19 pandemic (year 2021) on social anxiety (Mini-SPIN ≥ 6) among 13 to 20-year-old Finns compared to pre-pandemic years (2013 and 2015 combined) in females and males

		Females			Males	
		OR (95% CI)	*p*		OR (95% CI)	*p*
Total						
Model 1^1^: COVID-19 (year 2021)^2^		2.65 (2.60–2.69)	< 0.0001		1.53 (1.50–1.57)	< 0.0001
Model 2^1^: COVID-19 (year 2021)^2^		2.69 (2.64–2.74)	< 0.0001		1.56 (1.53–1.60)	< 0.0001
Model 3^1^: COVID-19 (year 2021)^2^		2.34 (2.29–2.38)	< 0.0001		1.53 (1.49–1.57)	< 0.0001
Age group 13–15 years						
Model 1^1^: COVID-19 (year 2021)^2^		2.64 (2.57–2.72)	< 0.0001		1.55 (1.50–1.61)	< 0.0001
Model 2^1^: COVID-19 (year 2021)^2^		2.71 (2.64–2.79)	< 0.0001		1.57 (1.51–1.63)	< 0.0001
Model 3^1^: COVID-19 (year 2021)^2^		2.37 (2.30–2.44)	< 0.0001		1.55 (1.49–1.61)	< 0.0001
Age group 16–17 years						
Model 1^1^: COVID-19 (year 2021)^2^		2.63 (2.56–2.70)	< 0.0001		1.53 (1.48–1.58)	< 0.0001
Model 2^1^: COVID-19 (year 2021)^2^		2.70 (2.62–2.77)	< 0.0001		1.55 (1.49–1.60)	< 0.0001
Model 3^1^: COVID-19 (year 2021)^2^		2.33 (2.26–2.39)	< 0.0001		1.51 (1.46–1.57)	< 0.0001
Age group 18–20 years						
Model 1^1^: COVID-19 (year 2021)^2^		2.52 (2.38–2.67)	< 0.0001		1.58 (1.46–1.70)	< 0.0001
Model 2^1^: COVID-19 (year 2021)^2^		2.61 (2.47–2.77)	< 0.0001		1.61 (1.49–1.74)	< 0.0001
Model 3^1^: COVID-19 (year 2021)^2^		2.24 (2.10–2.38)	< 0.0001		1.54 (1.42–1.67)	< 0.0001

Odds ratios (OR) and 95% Confidence Intervals (95% CI) from unadjusted and adjusted logistic regression analyses by age group

^1^Model 1: unadjusted, only COVID-19 term in the model; Model 2 (same model as presented in the main article): adjusted for age group (total models only), parental education, living with both parents, immigrant status and region; Model 3: in addition to model 2 adjustments, adjusted also for generalized anxiety (GAD-7 ≥ 10)

^2^Coded “1” for year 2021, “0” for years 2013 and 2015

**Table 7:** COVID-19-related measures in SHP 2021 in females and males

	Missing data %^1^	Females (*N* = 82,248)^2^		Males (*N* = 73,012)^2^		Gender difference
		%		%		*p*
Time spent in remote school during the school year	9.0					< 0.0001
Less than 1 month per semester		33.7		36.5		
1–2 months per semester at most		40.0		37.6		
Over 2 months in either semester		18.9		16.6		
Over 2 months in both semesters		7.4		9.3		
Support received in remote school for studies/learning	3.9^3^					< 0.0001
Not needed/received a lot		44.2		57.4		
Received some		36.5		32.5		
Not received, would have needed		19.3		10.0		
Worry, get infected, self	4.1					< 0.0001
Not at all		43.6		68.3		
Some		48.4		28.7		
A lot		8.0		3.0		
Worry, transmit to others	4.2					< 0.0001
Not at all		38.2		59.2		
Some		45.9		34.5		
A lot		15.9		6.2		
Effect on contacts with friends	4.4^4^					< 0.0001
No influence		47.1		61.9		
Decreased		38.0		27.0		
Increased		14.9		11.1		

^1^ Of those participating in 2021 fulfilling the inclusion criteria for the study, excluding suspected mischievous/unreliable answers (see methods), *N* = 155,260

^2^Suspected mischievous/unreliable answers excluded (see methods)

^3^In addition, 13.5% of cases were not in remote school at all and excluded from this variable

^4^In addition, 3.7% of cases answering “Does not apply to me” were excluded from this variable
