# Supplementary material for: Social anxiety in Finnish adolescents from 2013 to 2021: change from pre-COVID-19 to COVID-19 era, and mid-pandemic correlates
Source: Soc Psychiatry Psychiatr Epidemiol. 2023 Apr 24;59(1):121–36. doi: 10.1007/s00127-023-02466-4 (PMC10125255; doi:10.1007/s00127-023-02466-4)
Supplement: Supplementary file 1 — Supplementary file1 (DOCX 36 kb) [file 127_2023_2466_MOESM1_ESM.docx]

Appendix Figure 1. Percentages of those with clinical level social anxiety disorder symptoms (Mini-SPIN ≥ 6) among 13 to 20-year-old Finns in years 2013, 2015 and 2021 by gender
